# Factors affecting longitudinal trajectories of plasma sphingomyelins: the Baltimore Longitudinal Study of Aging

**DOI:** 10.1111/acel.12275

**Published:** 2014-10-24

**Authors:** Michelle M Mielke, Veera Venkata Ratnam Bandaru, Dingfen Han, Yang An, Susan M Resnick, Luigi Ferrucci, Norman J Haughey

**Affiliations:** 1 Department of Health Science Research and Neurology, Mayo ClinicRochester, MN, USA; 2 Department of Neurology, Johns Hopkins University School of MedicineBaltimore, MD, USA; 3 Department of Psychiatry, Johns Hopkins University School of MedicineBaltimore, MD, USA; 4 Intramural Research Program, National Institute on Aging, National Institutes of HealthBaltimore, MD, USA

**Keywords:** aging, sphingomyelin, dihydrosphingomyelin, human, longitudinal, sex differences

## Abstract

Sphingomyelin metabolism has been linked to several diseases and to longevity. However, few epidemiological studies have quantified individual plasma sphingomyelin species (identified by acyl-chain length and saturation) or their relationship between demographic factors and disease processes. In this study, we determined plasma concentrations of distinct sphingomyelin species in 992 individuals, aged 55 and older, enrolled in the Baltimore Longitudinal Study of Aging. Participants were followed, with serial measures, up to 6 visits and 38 years (3972 total samples). Quantitative analyses were performed on a high-performance liquid chromatography-coupled electrospray ionization tandem mass spectrometer. Linear mixed models were used to assess variation in specific sphingomyelin species and associations with demographics, diseases, medications or lifestyle factors, and plasma cholesterol and triglyceride levels. We found that most sphingomyelin species increased with age. Women had higher plasma levels of all sphingomyelin species and showed steeper trajectories of age-related increases compared to men. African Americans also showed higher circulating sphingomyelin concentrations compared to Caucasians. Diabetes, smoking, and plasma triglycerides were associated with lower levels of many sphingomyelins and dihydrosphingomyelins. Notably, these associations showed specificity to sphingomyelin acyl-chain length and saturation. These results demonstrate that longitudinal changes in circulating sphingomyelin levels are influenced by age, sex, race, lifestyle factors, and diseases. It will be important to further establish the intra-individual age- and sex-specific changes in each sphingomyelin species in relation to disease onset and progression.

## Introduction

The plasma lipidome is composed of numerous lipids that comprise specific variations in both structure and function. It has been increasing recognized that lipids are associated with the development and progression of many diseases, as well as aging and longevity (Vaarhorst *et al*., 2011; Yu *et al*., 2012; Gonzalez-Covarrubias *et al*., 2013). Sphingomyelins are among the most abundant lipids in many mammalian cells, tissues, and the circulation. In cells, sphingomyelins are central to the assemblage of lipid rafts and ordered membrane microdomains (Simons & Ikonen, 1997; Simons & Vaz, 2004; Quinn & Wolf, 2009). The biophysical properties of these lipids play important roles in the functional regulation of membrane-spanning proteins (Contreras *et al*., 2012) and in cholesterol homeostasis (Gatt & Bierman, 1980; Slotte & Bierman, 1988). Sphingomyelins are also rate-limiting precursors for other sphingolipids classes, such as ceramides, that are directly involved in a variety of cell-signaling events (Milhas *et al*., 2010). Niemann–Pick disease types A and B are genetic lipid storage disorders resulting from the lysosomal accumulation of sphingomyelin. Sphingomyelin metabolism has also been linked to other diseases including coronary artery disease (Jiang *et al*., 2000), ovarian endometriosis (Vouk *et al*., 2012), and cancer (Kim *et al*., 2013; Petersen *et al*., 2013), and also with longevity (Gonzalez-Covarrubias *et al*., 2013).

The majority of studies that examined sphingomyelins have focused on total sphingomyelin levels. However, there are a variety of sphingomyelin species (identified by acyl-chain length and saturation), with differential subcellular, cellular and tissue distributions that contribute to distinct cellular functions (Hannun & Obeid, 2011). With improved understanding of the role of the specific carbon-chain lengths in disease processes at the biochemical level, there is a need to translate this work to humans. There are little data on how plasma sphingomyelin and dihydrosphingomyelin levels are affected by age, sex, race, and changes in disease status within individuals or in the population. Previous, carefully validated studies of individual sphingomyelin species have been based on small samples of 10–15 people and young individuals (i.e., <50 years) (Hammad *et al*., 2010). It is important to extend this research to further quantify individual sphingomyelin species in larger populations to begin exploring factors that affect sphingomyelin concentrations in people over 50 years of age and to better understand the relationship between individual sphingomyelin species and disease risk.

In this study, we quantified individual species of plasma sphingomyelin and dihydrosphingomyelin in 992 individuals, aged 55 and older, enrolled in the Baltimore Longitudinal Study of Aging (BLSA). Individuals were followed, with serial measures, up to 6 visits and 38 years, allowing assessment of both interindividual variation and intra-individual changes over time in the sphingomyelins. In addition, we assessed factors associated with variation and changes in these levels including demographics, diseases (e.g., diabetes, hypertension), medications (e.g., statins), lifestyle factors (e.g., smok-ing, body mass index), and other blood lipids (e.g., cholesterol, triglycerides).

## Results

### Participant characteristics

The present analyses included 992 BLSA participants (366 women and 626 men) with at least 2 blood draws after the age of 55 years. The characteristics of the BLSA participants at the first blood draw included in the present analyses, by sex, are shown in Table1. Compared to men, women were slightly older (mean: 64.6 vs. 62.7 years, *P *=* *0.001), included a higher proportion of African Americans (18.3% vs. 9.3%, *P *<* *0.001), had fewer years of education (mean: 15.7 vs. 16.8, *P *<* *0.001), fewer depressive symptoms (mean: 4.7 vs. 5.7, *P *=* *0.006), lower waist–hip ratios (WHR; mean: 0.8 vs. 0.9, *P *<* *0.001), and higher total cholesterol (mean: 229.8 vs. 221.7, *P *=* *0.003). Women were less likely than men to have ever smoked (27.0% vs. 72.2%, *P *<* *0.001) or to have a diagnosis of prediabetes/diabetes (20.9% vs. 59.2%, *P *<* *0.001).

**Table 1:** Baseline characteristics of the Baltimore Longitudinal Study on Aging participants, by sex, at the first visit with a blood draw included in this study

Variable	Men		Women		*P*-value
	Total *N*	*N*(%)/mean (SD)	Total *N*	*N*(%)/mean (SD)	
Age	626	62.7 (7.9)	366	64.6 (8.4)	0.001
Race	626		366		<0.001
Caucasian		568 (90.7%)		299 (81.7%)	
African American		58 (9.3%)		67 (18.3%)	
Education	626	16.8 (2.7)	366	15.7 (2.5)	<0.001
Ever smoking	626	452 (72.2%)	366	172 (47.0%)	<0.001
Body mass index	624	26.2 (3.5)	366	25.7 (4.6)	0.062
Waist–hip ratio	486	0.9 (0.1)	355	0.8 (0.1)	<0.001
Depressive symptoms (CES-D)	622	5.7 (5.8)	364	4.7 (5.2)	0.006
Diagnosis of hypertension	626	178 (28.4%)	366	84 (23.0%)	0.059
Diabetes	626		366		<0.001
None		256 (40.9%)		286 (78.1%)	
Prediabetes		329 (52.6%)		67 (18.3%)	
Diabetes		41 (6.6%)		13 (2.6%)	
Chronic kidney disease	626	12 (1.9%)	366	13 (3.6%)	0.113
Any cancer	626	28 (4.5%)	366	18 (4.9%)	0.748
APOE e4 allele	464	120 (25.9%)	320	94 (29.4%)	0.278
Total cholesterol	595	221.7 (40.9)	351	229.8 (40.9)	0.003
Triglycerides	578	118.3 (80.1)	341	108.8 (81.1)	0.080
Statin use	626	37 (5.9%)	366	27 (7.4%)	0.364

The diagnoses of diabetes and prediabetes at each visit were established by combining information on medications, fasting glucose, and glucose levels at 2 h of a standard glucose tolerance test. In particular, participants who were taking antidiabetes medication or had a fasting glucose >126 mg dL^−1^ and/or a 2-h glucose >200 mg dL^−1^ were defined as diabetics. Among those who had no diabetes, participants with fasting glucose >100 mg dL^−1^ and/or a 2-h glucose >140 mg dL^−1^ were defined as having prediabetes. CESD, Center for Epidemiologic Studies Depression Scale.

### Plasma sphingomyelin concentrations by age and sex at baseline

The cross-sectional relationships of sphingomyelin and dihydrosphingomyelin concentrations with age and sex were similar across lipid species. In general, levels were higher in women and gradually increased with age for both men and women. Table2 provides the raw means and standard deviations (SD) of the individual sphingomyelin and dihydrosphingomyelin species at baseline, by 10-year-age group and sex. As there were only 7 individuals aged 85–94 years old at the first visit, these individuals were included with the 75- to 84-year-old group to create a 75 and older age group.

**Table 2:** Plasma sphingomyelins and dihydrosphingomyelins cross-sectionally by age and sex at the Baltimore Longitudinal Study of Aging visit in the present analyses

Plasma Sphingomyelins (ng mL^−1^ ×10^4^)	Aged 55–64 years			Aged 65–74 years			Aged ≥75 years		
	*N*	Mean (SD)	Range	*N*	Mean (SD)	Range	N	Mean (SD)	Range
Sphingomyelins									
C16:0									
Women	212	51.87 (14.97)	22.37–98.67	105	53.77 (13.81)	17.71–93.51	49	57.02 (17.56)	26.03–102.41
Men	431	51.19 (17.03)	10.67–117.20	127	53.72 (16.17)	17.41–109.62	67	51.00 (15.68)	21.66–100.43
C18:0									
Women	212	7.76 (2.90)^c^	2.42–18.77	105	8.24 (2.72)^b^	2.31–15.62	48	8.29 (3.14)^b^	2.87–18.67
Men	430	6.84 (2.63)	1.30–15.63	126	7.30 (2.66)	2.09–15.35	67	6.94 (1.98)	3.42–13.06
C20:0									
Women	211	8.63 (3.10)^b^	3.02–21.74	105	9.13 (2.71)	4.09–15.27	49	9.12 (3.22)	4.26–18.41
Men	431	7.97 (2.86)	1.24–21.41	127	8.87 (3.27)	3.13–19.16	67	8.12 (2.31)	3.03–12.86
C22:0									
Women	210	11.83 (5.02)	3.20–33.39	105	12.13 (4.15)	3.91–23.12	49	12.37 (5.12)	3.15–24.69
Men	429	11.59 (5.08)	2.03–32.77	127	12.97 (5.74)	3.88–29.28	67	11.9 (4.42)	5.06–25.42
C24:0									
Women	210	10.08 (4.57)	3.07–28.39	105	10.53 (4.09)	3.97–22.64	49	10.34 (4.22)	2.37–22.18.00
Men	429	9.94 (4.46)	2.07–28.99	127	10.89 (4.58)	3.26–24.23	67	10.3 (4.21)	4.26–27.48
C16:1									
Women	212	7.10 (2.20)^c^	2.60–15.43	105	7.54 (2.45)^a^	2.77–14.52	49	7.49 (2.15)^c^	4.56–15.00
Men	431	6.15 (2.04)	1.48–14.90	127	6.78 (2.07)	2.77–13.83	67	6.24 (1.66)	2.22–10.49
C18:1									
Women	209	20.37 (8.18)^c^	4.78–51.99	105	20.88 (7.34)	5.38–45.55	47	20.86 (8.72)	6.93–46.92
Men	429	17.41 (7.48)	4.5–50.02	125	20.52 (8.55)	6.03–45.35	67	18.29 (6.66)	8.94–45.71
C20:1									
Women	212	3.57 (1.58)^c^	1.33–8.79	105	3.73 (1.54)^a^	0.82–8.87	49	3.93 (1.64)^c^	1.98–9.06
Men	431	2.83 (1.15)	0.63–8.07	127	3.35 (1.32)	1.15–7.38	67	3.04 (1.02)	1.17–6.46
C22:1									
Women	211	12.96 (4.98)^c^	3.39–28.62	105	13.34 (4.67)	3.33–27.34	49	14.3 (5.95)^b^	6.66–32.59
Men	431	11.05 (4.58)^a^	1.87–34.05	127	12.28 (5.02)	3.74–31.62	67	11.61 (4.4)	4.51–22.71
C24:1									
Women	211	24.87 (9.77)	10.97–64.34	105	26.63 (9.03)	9.97–52.56	49	27.56 (12.02)	12.37–67.73
Men	431	22.98 (9.84)	6.22–69.00	127	26.29 (10.16)	10.07–68.90	67	25.1 (8.65)	10.87–54.87
DihydoSphingomyelins									
C16:0									
Women	211	1.08 (0.44)^b^	0.26–2.66	105	1.11 (0.47)	0.36–2.70	49	1.27 (0.46)^c^	0.46–2.25
Men	432	0.96 (0.43)	0.13–2.47	127	1.00 (0.40)	0.27–2.23	67	0.99 (0.39)	0.37–2.59
C18:0									
Women	212	21.40 (8.16)	4.07–50.10	105	22.03 (8.18)	7.08–54.52	49	22.59 (7.45)	7.74–39.72
Men	431	20.13 (8.09)	1.53–55.98	126	24.04 (9.42)	0.96–58.40	67	24.73 (8.86)	11.37–55.04
C22:0									
Women	211	0.74 (0.38)	0.18–2.06	102	0.79 (0.35)	0.27–1.95	48	0.80 (0.45)	0.17–2.13
Men	428	0.73 (0.38)	0.14–2.09	127	0.76 (0.42)	0.08–2.20	66	0.76 (0.37)	0.25–1.77
C24:0									
Women	209	1.70 (0.70)	0.57–4.46	105	1.85 (0.71)	0.64–4.00	49	1.78 (0.77)	0.53–4.60
Men	428	1.69 (0.74)	0.41–4.52	127	1.78 (0.73)	0.61–4.70	67	1.71 (0.69)	0.63–4.18

T-tests used to compare cross-sectional sex differences within each age group.

a *P* < 0.05

b *P* < 0.01

c *P* < 0.001.

### Longitudinal intra-individual stability

Longitudinally, the mean (SD) number of plasma serial samples per person with measured sphingomyelins and dihydrosphingomyelins was 4.0 (0.7). These samples were collected over a mean of 14.3 years (6.7) with a range of 2.0–38.9 years and included 3972 total samples from the 992 participants. The intraclass correlation (ICC) of the various sphingomyelin species, a measure of how well different molecular classes track over time, ranged from 0.23 for C24:0 to 0.38 for 20:1 (all *P *<* *0.0001). The ICC of dihydrosphingomyelins was lower and ranged from 0.13 for C22:0 to 0.27 for C18:0 (all *P *<* *0.0001).

### Variables cross-sectionally and longitudinally associated with the sphingomyelins and dihydrosphingomyelins

**Figure 1:**
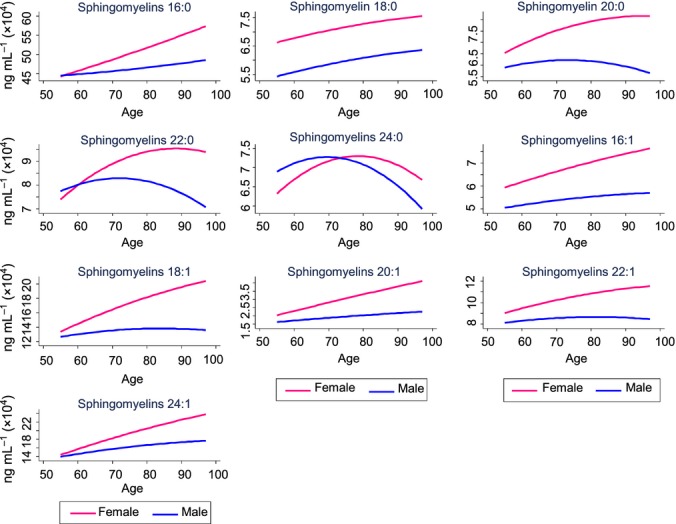
Plasma sphingomyelins by age and sex. Concentrations are based on predicted values obtained in linear mixed models and controlling for additional factors specific to each model (see Table3).

**Figure 2:**
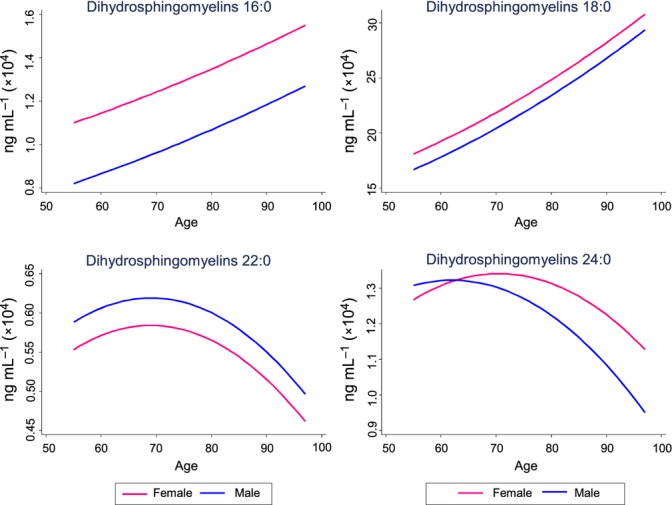
Plasma dihydrosphingomyelins by age and sex. Concentrations are based on predicted values obtained in linear mixed models and controlling for additional factors specific to each model (see Table4).

We next identified factors associated cross-sectionally and longitudinally, in a time-dependent manner, with each of the sphingomyelin and dihydrosphingomyelin species using linear mixed models. We identified the most parsimonious models and determined the variables most strongly associated with each sphingomyelin class and individual species. Table3 and Fig.1 provide the model and model-fitted values for the sphingomyelins; Table4 and Fig.2 provide the model and model-fitted values for the dihydrosphingomyelins.

**Table 3:** Variables cross-sectionally and longitudinally associated with specific carbon-chain lengths of plasma sphingomyelins in the Baltimore Longitudinal Study of Aging

Covariates	C16:0		C18:0		C20:0		C22:0		C24:0		C16:1		C18:1		C20:1		C22:1		C24:1	
	Baseline	Time-Dependent	Baseline	Time-Dependent	Baseline	Time-Dependent	Baseline	Time-Dependent	Baseline	Time-Dependent	Baseline	Time-Dependent	Baseline	Time-Dependent	Baseline	Time-Dependent	Baseline	Time-Dependent	Baseline	Time-Dependent
	b (SE)	b (SE)	b (SE)	b (SE)	b (SE)	b (SE)	b (SE)	b (SE)	b (SE)	b (SE)	b (SE)	b (SE)	b (SE)	b (SE)	b (SE)	b (SE)	b (SE)	b (SE)	b (SE)	b (SE)
Age	2999.3 (467.4)^c^		250.6 (67.9)^c^		505.8 (95.9)^c^		705.2 (154.6)^c^		301.8 (132.3)^a^		432.3 (61.4)^c^		1842.5 (269.6)^c^		503.5 (45.7)^c^		686.6 (140.7)^c^		2399.6 (209.9)^c^	
Age2	7.1 (22.8)		−2.3 (3.7)		−9.6 (4.9)^a^		−18.0 (8.0)^a^		−16.8 (7.1)^a^		−2.3 (3.2)		−13.9 (12.3)		−0.8 (2.3)		−7.7 (7.5)		−12.1 (15.9)	
Men	−29 876.0 (5524.5)^c^	−2133.2 (518.2)^c^	−12 029.1 (983.9)^c^		−12 861.4 (1560.9)^c^	−447.7 (114.8)^c^	−5795.5 (2646.6)^a^	−640.6 (182.6)^c^	1207.8 (1757.0)	−328.4 (156.7)^a^	−12 555.1 (825.9)^c^	−249.5 (73.9)^b^	−28 973.9 (3490.9)^c^	−1456.4 (300.6)^c^	−9276.8 (638.3)^c^	−346.0 (55.3)^c^	−16 747.3 (1905.1)^c^	−511.9 (169.9)^b^	−24 883.6 (4197.3)^c^	−2375.7 (375.3)^c^
African American	33 328.5 (7756.3)^c^		6533.9 (1440.4)^c^		14 383.6 (1751.2)^c^		31 652.9 (3032.6)^c^		25 874.7 (2552.3)^c^		7225.1 (1165.0)^c^		44 152.1 (4969.8)^c^		10 288.8 (886.3)^c^		27 408.7 (2770.8)^c^		72 392.8 (5 852.1)^c^	
APOE e4	10 077.7 (5558.3)										1408.9 (840.6)						4492.9 (1999.9)^a^			
BMI	−2252.1 (622.3)^c^		670.2 (110.5)^c^		281.0 (143.4)^a^	37.2 (13.8)^b^	488.4 (245.5)^a^	76.2 (22.4)^b^	−46.4 (201.4)	50.5 (19.3)^b^	515.3 (92.1)^c^		2187.0 (386.4)^c^		307.6 (69.5)^c^	12.1 (6.6)	584.4 (218.9)^b^	44.1 (20.8)^a^	−125.7 (461.8)	
WHR					14 600.5 (7576.9)		24 729.5 (12 955.2)													
CESD					−162.2 (78.6)^a^		−312.6 (134.2)^a^		−212.6 (117.2)											
Pre-diabetes	−10 584.3 (5269.9)^a^	−1103.3 (525.0)^a^			−2702.3 (1133.4)^c^		−5419.4 (1934.7)^b^		−3784.1 (1661.4)^a^		−3543.1 (756.9)^c^		−7800.1 (2981.2)^b^		−2260.9 (541.6)^c^		−6422.3 (1793.6)^c^		−9210.8 (3790.0)^b^	
Diabetes	−3407.1 (8011.4)	11.7 (763.5)			−852.1 (1705.4)		−3255.3 (2894.5)		−1786.5 (2463.1)		−2284.5 (1131.8)^a^		−12 099.5 (4598.0)^b^		−2430.0 (830.1)^b^		−9143.3 (2675.9)^b^		−11 511.9 (5721.2)^a^	
Statin use	−11 090.0 (6407.5)	−1330.1 (703.6)			6841.7 (1322.3)^c^		6988.9 (2264.5)^b^				2266.4 (890.9)^a^		9655.4 (3398.7)^b^		3792.2 (623.3)^c^		6493.1 (2108.5)^b^		19 530.4 (4445.6)^c^	
Hypertension													1186.4 (2991.2)	476.7 (284.9)						
Cancer					4261.5 (1588.0)^b^				4635.9 (2591.9)	−501.5 (245.0)^a^	2831.1 (1057.0)^b^				2267.3 (771.5)^b^				10 135.1 (5374.5)	
Smoker	8370.5 (4401.4)		1750.9 (816.2)^a^	−186.4 (74.3)^a^	2125.8 (1002.7)^a^						1539.8 (663.4)^a^				1034.7 (502.7)^a^				6155.4 (3352.9)	
Cholesterol	1576.5 (62.4)^c^		199.7 (10.6)^c^		218.6 (13.4)^c^		334.4 (22.8)^c^		322.2 (19.2)^c^		155.1 (9.0)^c^	1.7 (0.9)^a^	474.6 (35.3)^c^		49.2 (6.4)^c^		338.5 (21.3)^c^		570.8 (44.9)^c^	
Triglycerides	−283.4 (34.0)^c^		13.4 (5.9)^a^		−12.0 (7.2)		−41.8 (12.4)^b^		−42.4 (10.6)^c^		−17.4 (5.0)^b^	−0.9 (0.5)	−43.7 (18.8)^b^		−6.9 (3.4)^a^		−65.9 (11.5)^c^		−133.2 (24.0)^c^	

APOE e4, presence of at least one APOE E4 allele; BMI, body mass index; WHR, waist–hip ratio

We forced age, age 2, sex, and BMI into all models then used backward selection with *P* < 0.10 to determine which variables to include in the final model of each sphingomyelin species and carbon-chain length. ^a^*P* < 0.05; ^b^*P* < 0.01; ^c^*P* < 0.001.

**Table 4:** Variables cross-sectionally and longitudinally associated with plasma dihydrosphingomyelins in the Baltimore Longitudinal Study of Aging

	C16:0		C18:0		C22:0		C24:0	
	Baseline	Time-Dependent	Baseline	Time-Dependent	Baseline	Time-Dependent	Baseline	Time-Dependent
Covariates	b (SE)	b (SE)	b (SE)	b (SE)	b (SE)	b (SE)	b (SE)	b (SE)
Age	101.3 (15.6)^c^		2785.5 (281.8)^c^		−3.0 (6.4)		3.2 (20.3)	
Age 2	0.5 (0.7)		19.2 (15.6)		−1.6 (0.6)^b^		−3.0 (1.1)^b^	
Men	−2795.9 (230.0)^c^		−14 287.7 (4130.0)^b^		350.3 (125.1)^b^		−376.2 (261.6)	−51.6 (24.2)^a^
African American			19 115.4 (5822.6)^b^		1007.3 (186.0)^c^		3037.0 (378.9)^c^	
BMI	48.8 (21.0)^b^		668.9 (454.1)		−13.4 (14.7)	2.9 (1.5)	−42.3 (30.3)	
WHR	2444.9 (1135.5)^a^							
Prediabetes	−232.4 (169.3)	−42.4 (16.6)^a^	−10 907.2 (3611.8)^b^	−725.1 (353.4)^a^			−492.9 (255.3)	
Diabetes	−52.5 (258.9)	−47.3 (24.3)	−8605.3 (5551.7)	−1136.3 (522.0)^a^			−273.1 (399.6)	
Statin use			8238.3 (4166.0)^a^					
Cancer	533.7 (237.8)^c^		24 814.7 (5535.4)^c^	−1517.3 (544.6)^b^			213.1 (399.6)	−79.4 (38.3)^a^
Smoker	205.1 (148.3)	−28.1 (14.2)^a^						
Cholesterol	17.6 (2.0)^c^		282.0 (42.3)^c^		29.8 (1.5)^c^		62.4 (3.0)^c^	
Triglycerides	4.5 (1.1)^c^		130.2 (22.5)^c^		−2.8 (0.8)^b^		−6.3 (1.7)^c^	0.3 (0.2)

BMI, body mass index; WHR, waist–hip ratio.

We forced age, age 2, sex, and BMI into all models then used backward selection with *P* < 0.10 to determine which variables to include in the final model of each dihydrosphingomyelin carbon-chain length. ^a^*P* < 0.05; ^b^*P* < 0.01; ^c^*P* < 0.001.

### Associations with demographic variables

Similar to the unadjusted analysis, after controlling for covariates, all sphingomyelins increased with age within individuals (Table3, Fig.1). However, the age-squared term of the longer sphingomyelin acyl-chain lengths including C20:0 (*b* = −9.6, *P *=* *0.049), C22:0 (*b* = −18.0, *P *=* *0.024), and C24:0 (*b* = −16.8, *P *=* *0.018) indicated a biphasic relationship in which the age-related increase reached a plateau and then decreased with advanced age (see Fig.1). Among dihydrosphingomyelins, only C16:0 and C18:0 significantly increased with age (Table4, Fig.2). Women had higher baseline levels of almost all sphingomyelins and dihydrosphingomyelins and the sex by age interaction indicated that women also had greater increases with age. The only exception was dihydrosphingomyelin C24:0, which was higher in men at baseline and throughout the follow-up period. African Americans had significantly (*P *<* *0.001) higher levels of all sphingomyelins and almost all dihydrosphingomyelins throughout the follow-up.

### Associations with anthropometric and behavioral factors

In general, higher body mass index (BMI) was associated with higher levels of all sphingomyelins at baseline. There were also interactions with age for C20:0, C22:0, and C24:0, suggesting that individuals with higher BMI had greater age-related increases in these acyl-chain lengths. The only exception was sphingomyelin C16:0 in which high BMI was associated with lower sphingomyelins (*b* = −2176.4, *P *<* *0.001). A higher BMI was associated with higher dihydrosphingomyelins C16:0 and C18:0, but with lower C22:0 and C24:0 levels. Compared to BMI, there were fewer associations between WHR and either sphingomyelins or dihydrosphingomyelins. Ever smokers had higher sphingomyelin levels and also higher dihydrosphingomyelin C16:0 levels. Higher depressive symptoms were associated with lower concentrations of sphingomyelins C20:0 and C22:0. There were no associations between depressive symptoms and dihydrosphingomyelins or between antidepressant use and either sphingomyelins or dihydrosphingomyelins.

### Associations with blood cholesterol, triglycerides, statins, and APOE e4 genotype

There were positive associations (all *P *<* *0.001) between cholesterol and each sphingomyelin and dihydrosphingomyelin species. As the interactions between cholesterol and age were not significant, the strength of the association did not change with increasing age (Tables3 and 4). In contrast to cholesterol, associations between the sphingomyelins and triglycerides were primarily in the opposite direction. Higher concentrations of sphingomyelin C18:0 and dihydrosphingomyelins C16:0 and C18:0 were associated with higher triglycerides. However, higher levels of all other sphingomyelin and dihydrosphingomyelin species were associated with lower triglycerides. Similar to cholesterol, the relationship between sphingomyelins and triglycerides did not change with increasing age. Statin use at baseline was associated with higher levels of all sphingomyelins, except for C16:0 which was lower in statin users. However, the proportion of individuals taking statins at baseline was small and <6%. Statin use was also associated with higher dihydrosphingomyelin C18:0 at baseline. Individuals with at least one APOE e4 allele had a higher concentration of sphingomyelin C22:1 (*b* = 4492.9, *P *=* *0.025), but APOE genotype was not significantly associated with other sphingomyelin or dihydrosphingomyelin species.

### Associations with specific diseases

In multivariate models, individuals with either prediabetes or diabetes at baseline had significantly lower levels of all sphingomyelins, except C18:0, and dihydrosphingomyelins C16:0 and C18:0 (Tables3 and 4). Individuals with a self-reported history of cancer had higher levels of most sphingomyelins and dihydrosphingomyelins, but we were not able to break down the analyses into specific types of cancer. There were no significant associations between any sphingomyelin or dihydrosphingomyelin species and hypertension.

## Discussion

It has been increasingly recognized that lipids are associated with the development and progression of many diseases, as well as aging and longevity (Vaarhorst *et al*., 2011; Yu *et al*., 2012; Gonzalez-Covarrubias *et al*., 2013). Few large-scale clinical and epidemiological studies have examined plasma levels of sphingomyelins and dihydrosphingomyelins. Exploring the ‘normal’ ranges of plasma sphingomyelins and dihydrosphingomyelins by age and sex is important to study whether information of these specific lipid species is useful to assess the risk of many complex diseases that affect older persons and to gain insight into their pathophysiology. In the present study, we quantified plasma levels of individual sphingomyelin and dihydrosphingomyelin species (defined by acyl-chain lengths) in participants of the Baltimore Longitudinal Study of Aging. This included the examination of 14 total species of sphingomyelins and dihydrosphingomyelins and multiple outcomes. While this led to multiple comparisons, this step is critical because the carbon-chain lengths of a lipid species may be differentially associated with specific functions and diseases. In this initial descriptive study, we examined factors previously reported to be associated with each of these lipids to replicate previous findings and to ascertain longitudinal associations over an age-range when chronic diseases start to emerge.

We found that plasma sphingomyelin concentrations significantly varied with age, sex, and race. Almost all sphingomyelin species increased with age. Cross-sectionally, women had higher plasma sphingomyelin levels than men, which is consistent with previous reports (Nelson *et al*., 2006; Hammad *et al*., 2010; Gonzalez-Covarrubias *et al*., 2013; Weir *et al*., 2013). Plasma levels of most sphingomyelins showed a steeper trajectory of longitudinal increase with age in women compared to men. In addition, there were significant race differences, indicating elevated plasma concentrations in African Americans compared to Caucasians for all species. These data are consistent with findings from the Multi-Ethnic Study of Atherosclerosis which reported higher sphingomyelin levels in African Americans, Hispanics, and Chinese Americans compared to Caucasians (Nelson *et al*., 2006).

Higher levels of total cholesterol were associated with higher sphingomyelin concentrations of all species, consistent with other studies (Nelson *et al*., 2006; Yeboah *et al*., 2010; Gonzalez-Covarrubias *et al*., 2013). In contrast to cholesterol, higher triglyceride levels were associated with lower levels of all sphingomyelins except C18:0. This is consistent with our disease-related findings of lower sphingomyelins in diabetics as they tend to have higher triglyceride levels. Our finding is interesting in light of recent studies showing that higher levels of sphingomyelin and lower levels of triglycerides in midlife, especially among women, are associated with healthy aging and longevity (Vaarhorst *et al*., 2011; Yu *et al*., 2012; Gonzalez-Covarrubias *et al*., 2013).

Very few studies to date have examined plasma dihydrosphingomyelin levels. Dihydrosphingomyelin interacts with higher affinity to cholesterol than acyl-matched sphingomyelins. This causes increased stability and packing of the plasma membranes, leading to more rigid lipid microdomains (Kuikka *et al*., 2001; Vieira *et al*., 2010). These biophysical effects can modify the location and function of membrane-located receptors and signaling proteins with implications for subsequent signaling events [see (Haughey *et al*., 2010) for a further discussion of sphingolipids and cellular signaling]. Dihydrosphingomyelin levels have previously been shown to increase with age in the human lens and have been associated with cognitive decline in patients with Alzheimer’s disease (Deeley *et al*., 2010; Mielke *et al*., 2011). In this study, we also found that plasma dihydrosphingomyelin levels, especially the most abundant C16:0 and C18:0 acyl-chain lengths, increased with age. However, the longer acyl length, C22:0 and C24:0 did not. Indeed, other associations were opposite for the long chain-length dihydrosphingomyelins (C16:0 and C18:0), compared to the very long chain-length dihydrosphingomyelins (C22:0 and C24:0), in relation to BMI and triglycerides. To our knowledge, this is the first report describing the contrasting relationship between dihydrosphingomyelin species with demographic and disease variables and warrants additional research.

Limitations of the study warrant consideration. First, the BLSA is a community-dwelling volunteer cohort that is predominantly white (∼80%), of upper-middle socioeconomic status, and with an above-average educational level. While this may hinder generalizability, the relative homogeneity of the sample may be seen as an asset because the majority of individuals have good access to medical care and have remained relatively healthy over the follow-up interval. Second, we quantified sphingomyelins and dihydrosphingomyelins in the plasma and did not examine the composition of lipids by lipoprotein. Sphingomyelins and dihydrosphingomyelins are carried on multiple lipoproteins in the blood, and composition and quantification of the lipids on the specific lipoproteins may differ by age and with disease onset. For example, a recent study suggested that the sphingomyelin content of high-density lipoproteins (HDL) increased with age and hypothesized that changes in HDL composition could be linked to changes in the atherogenic properties of HDL (Holzer *et al*., 2013). However, to quantitate all of the lipids by specific lipoproteins would require many more runs and would take a significantly greater amount of time and effort. Thus, the present work is the first step in beginning to understand the relationship between plasma sphingomyelins and dihydrosphingomyelins with age and disease processes. Lastly, we did not examine the relationship of these lipids with vascular diseases including myocardial infarction and atherosclerosis. Previous studies have examined this association with conflicting results (Jiang *et al*., 2000; Nelson *et al*., 2006; Schlitt *et al*., 2006; Yeboah *et al*., 2010; Fernandez *et al*., 2013). The associations are complex and will involve many more analyses, so we will pursue and present this work in another paper.

Despite these limitations, there are also advantages to our study. First, we incorporated a large, well-characterized sample set with a longitudinal follow-up. Most studies examining sphingomyelin changes with age or disease have used cross-sectional cohorts (e.g., Yu *et al*., 2012). In the present study, we measured sphingomyelins and dihydrosphingomyelins among 992 BLSA participants (366 women and 626 men) with at least 2 blood samples (mean number was 4) over a 38-year time period. This study design allowed us to examine within-individual trajectories of age-related changes in plasma sphingomyelin levels. Second, studies of sphingomyelins and healthy aging often exclude individuals with several diseases (e.g., diabetes, hypertension). While this design is important for understanding changes in lipid profiles associated with longevity, it does not allow for the study of disease processes on these lipid levels. In the present study, we did not exclude individuals based on their medical history or risk factor status but controlled for these factors in investigating associations with age, sex, and race. Lastly, many studies of sphingomyelins have only reported total sphingomyelins, a sum of all carbon-chain lengths. The quantification of the individual chain lengths is especially important in understanding the mechanistic pathways influenced by selected molecular species. A recent paper highlighted distinct cellular functions for the different ceramide carbon-chain lengths and hydroxylations in yeast (Montefusco *et al*., 2013). As ceramides are precursors and metabolites of sphingomyelin, it is not surprising that we also found that the relationship between the plasma sphingomyelins and potential modifiers varied by carbon-chain length. While the measurement of total sphingomyelins may be easier, much information may be missed by not examining the specific carbon-chain lengths.

## Experimental procedures

### Participants

This study uses samples and data from the Baltimore Longitudinal Study of Aging (BLSA). Initiated in 1958, the BLSA is a longitudinal cohort study of community-dwelling individuals aimed at examining the physiological and psychological aspects of human aging (Shock *et al*., 1984). At each study visit, participants underwent an extensive medical examination, neuropsychological battery, blood draw, medical history, and medication review. Historically, BLSA visits occurred every 2 years. In 2003, the sampling times were modified because historical data indicated nonlinear changes at the oldest ages. To improve sampling of the epoch with accelerated physical and cognitive change, individuals aged 80 and older have been evaluated annually since 2003. The protocol was approved by the local institutional review board, and written informed consent was obtained prior to participation at each assessment.

Blood samples were drawn at all visits from the antecubital vein between 7 and 8 AM after an overnight fast as described (Shock *et al*., 1984). Participants were not allowed to smoke, engage in physical activity, or take medications before the sample was collected. Plasma samples were immediately processed, cataloged, and stored at −80 °C.

### Description of variables examined in relation to lipid levels

All variables were assessed at baseline and each follow-up visit using the same method. Demographic variables included age, sex, race, and years of education. Height (in meters) and weight (in kilograms) were measured for all participants. Body mass index (BMI) was determined as kilograms per meter squared. Waist–hip ratio (WHR) was measured in the standing position, as previously reported (Shimokata *et al*., 1989), using a flexible metal tape. Smoking status was determined by a questionnaire that classified each individual as an ever or never smoker. Depressive symptoms were assessed with the Center for Epidemiologic Studies Depression Scale (CES-D). Medical histories were recorded that included hypertension, cancer, and chronic kidney disease (CKD). The diagnoses of diabetes and prediabetes were established by combining information on medications, fasting glucose, and glucose levels at 2 h of a standard glucose tolerance test. In particular, participants who were prescribed antidiabetes medication or with a fasting glucose >126 mg dL^−1^ and a 2-h glucose >200 mg dL^−1^ were defined as diabetes. Among those who had no diabetes, participants with fasting glucose >100 mg dL^−1^ and/or a 2-h glucose >140 mg dL^−1^ were defined as having prediabetes. Medication use was verified at each visit.

Plasma total cholesterol and triglycerides were determined by an enzymatic method (Abbott Laboratories ABA-200 ATC Biochromatic Analyzer, Irving, TX, USA). Apolipoprotein E (APOE) genotype was determined in approximately half of the sample set using polymerase chain reaction amplification of leukocyte deoxyribonucleic acid followed by HhaI digestion and product characterization (Hixson & Vernier, 1990) and by TaqMan in the remaining half, relying on several single nucleotide polymorphisms around the APOE gene (Koch *et al*., 2002).

### Lipid extraction and LC/ESI/MS/MS analysis

**Figure 3:**
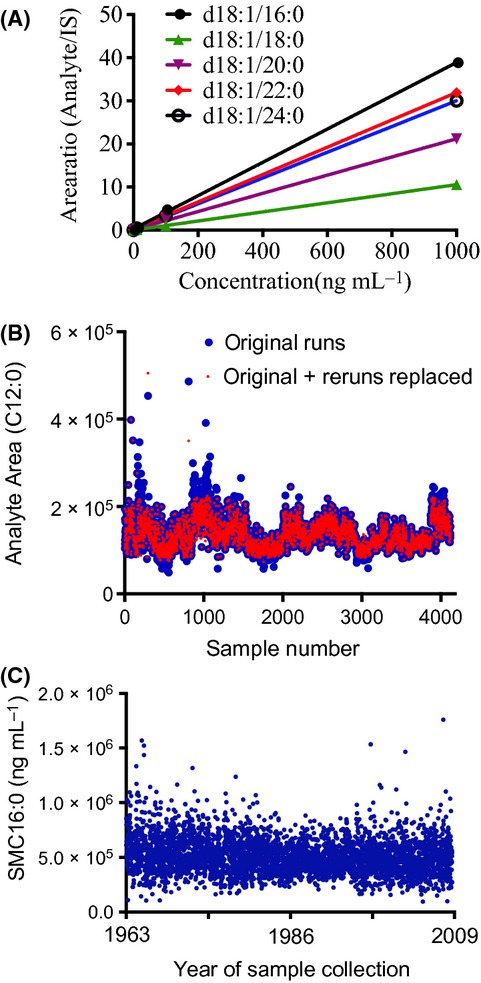
(A) Five-point standard curves for the indicated SM species. (B) Initial and rerun data showing values obtained for the internal standard SM C12:0. (C) SM C16:0 scatterplot by date of visit showing a random distribution, suggesting that SM was stable during long-term storage of plasma.

A crude lipid extraction of plasma was obtained using a modified Bligh and Dyer procedure as previously described (Haughey *et al*., 2004; Bandaru *et al*., 2013). Sphingomyelin C12:0 (1.3 μg mL^−1^; Avanti Polar Lipids, Alabaster, Alabama) was included in the extraction solvent as an internal standard. The chloroform layer containing a crude lipid extract was dried in a nitrogen evaporator (Organomation Associates Inc., Berlin, MA, USA) and stored at −80 °C. Dried extracts were resuspended in pure methanol just prior to analysis. Analyses of sphingomyelins were performed on a triple quadrupole mass spectrometer (API3000, AB Sciex Inc., Thornhill, Ontario, Canada) using instrument parameters similar to those described in previous studies (Bandaru *et al*., 2007, 2011, 2013). Samples were injected using an Agilent 1100 high-pressure liquid chromatography (HPLC) (Agilent Technologies, Inc., Santa Clara CA, USA) equipped with a reverse phase C18 column (Phenomenex, Torrance, CA, USA). Sphingomyelin species were separated by gradient elution at the flow rate of 0.7 mL min^−1^. Mobile phases consisted of A: 60% methanol, 39% H_2_O, 1% formic acid with 5 mm ammonium formate; and B: 99% methanol, 1% formic acid, and 5 mm ammonium formate. Gradient conditions were as follows: 60% B for 0.01 min, a gradual increase to 100% B over the next 0.49 min, and hold at 100% B for 3 min. Decline from 100% to 0% B during the next 0.01 min, hold at 0% B for 0.99 min, increase from 0% to 60% B for 0.5 min and hold at 60% B for the final 0.5 min. The eluted sample was injected into the ion source where the detection of each sphingomyelin species was conducted by multiple reaction monitoring (MRM) in positive mode. Detailed MRM transitions of individual molecular species for sphingomyelin precursor and fragment ions are provided in Table5. The ion spray voltage (V) was 5500 at a temperature of 80 °C with a nebulizer gas of 9 psi, curtain gas of 8 psi, and the collision gas set at 10 psi. The declustering potential was 60 V, the focusing potential 300 V, the entrance potential 10 V, the collision energy 30 V, and the collision cell exit potential 10 V. MS/MS scanned from 300 to 1000 atomic mass units (amu) per second with steps of 0.1 amu. Eight-point calibration curves (0.1 to 1000 ng mL^−1^) were constructed by plotting the area under the curve for sphingomyelin C16:0, C18:0, C20:0, C22:0, and C24:0 (Avanti polar lipids, Alabaster, AL, USA) prepared in pure methanol normalized to the C12:0 internal standard. The correlation coefficients (*R*^2^) obtained were >0.999 (Fig.3A). Concentrations of sphingomyelins in each sample were determined by fitting the identified sphingomyelin and dihydrosphingomyelin species to these standard curves based on acyl-chain length (i.e., concentrations of C16:0 and C16:1 were both determined based on the C16:0 standard curve). Instrument efficiency was closely monitored using a series of internal standards run daily. Area under the curve for these standards was plotted weekly to track instrument efficiency. Runs were stopped when consistent deviations of more than 30% from the median internal standard values were noted, and the instrument was recalibrated. Individual samples in which the internal standard deviated more than 30% of the median internal standard value were rerun. Using these criteria, approximately 20% of samples required reanalysis. Data that produced internal standard values closest to the overall median value were incorporated in the final analysis (Fig.3B). Instrument control and quantification were performed using Analyst 1.4.2 and MultiQuant software (AB Sciex Inc., Thornhill, ON, Canada). Intra-day coefficient of variation (CV) was determined by analyzing 5 pooled samples 6 times. Intra-day CVs were as follows: C16:0 (2.1%), C18:0(5.7%), C20:0 (2.1%), C22:0 (3.0%), C24:0 (3.6%), C16:1 (2.1%), C18:1 (3.6%), C20:1 (9.6%), C22:1 (4.0%), C24:1 (2.4%) and dihydrosphingomyelins (DHSM) C16:0 (4.0%), C18:0 (3.7%), C22:0 (7.4%), and C24:0 (8.6%). Inter-day CVs were determined using five repeat measurements of 10 pooled samples over a 3-month period (the time required for analysis of all study samples). Interday CVs for sphingomyelin species were as follows: C18:0/C16:0 (12%), C18:0/C18:0 (20%), C18:0/C20:0 (24%), C18:0/C22:0 (12%), C18:0/C24:0 (8%),C18:0/C16:1 (12%), C18:0/C18:1 (16%), C18:0/C20:1 (20%), C18:0/C22:1 (28%), C18:0/C24:1 (12%),C18:1/C16:0 (14%), C18:1/C18:0 (8%), and C18:1/C24:0 (6%). Recovery was determined by comparing C12:0 internal standard levels in extracted plasma to equal amounts of C12:0 sphingomyelin prepared in pure methanol. The average extraction recovery obtained was 93%. As the dates of sample collection for this study ranged from 1968 to 2009 and were run in random order over the 3 months, we estimated sample stability by arranging data for each sphingomyelin species by date of study visit. We reasoned that sample degradation or continued enzymatic activity would be manifest by increasing or decreasing trends in analyte concentrations coincident with storage time. When arranged by date of visit, each species showed a random scatter (Fig.3C shows sphingomyelin C16:0), suggesting that sphingomyelin content of plasma was stable with long-term −80 °C storage.

**Table 5:** Multiple reaction monitoring (MRM) transitions for molecular species of sphingomyelin precursor and fragment ions

Sphingomyelins	Precursor/fragment ion m/z
d18:1/12:0	647.7/184.4*
d18:1/16:0	703.8/184.4
d18:1/18:0	732.1/184.4
d18:1/20:0	759.8/184.4
d18:1/22:0	787.9/184.4
d18:1/24:0	815.9/184.4
d18:1/26:0	843.9/184.4
d18:1/16:1	701.5/184.4
d18:1/18:1	729.4/184.4
d18:1/20:1	757.4/184.4
d18:1/22:1	785.4/184.4
d18:1/24:1	813.9/184.4
d18:1/26:1	841.9/184.4

Dihydrosphingomyelins	Precursor/fragment ion m/z
d18:0/16:0	706.9/184.4
d18:0/18:0	734.8/184.4
d18:0/20:0	761.8/184.4
d18:0/22:0	789.9/184.4
d18:0/24:0	817.9/184.4
d18:0/26:0	845.9/184.4

* Internal standard.

### Statistical analyses

Sex differences in baseline demographic and health-related characteristics were examined using Fisher’s exact test for categorical variables and *t*-tests or ANOVA for continuous variables. We examined all sphingomyelin species for outliers. As normal ranges for sphingomyelins in plasma are not yet known, we conservatively defined an outlier as any sphingomyelin species with a concentration of more than 3 interquartile ranges (IQR, 25th percentile–75th percentile) from the median. Within each class of the plasma sphingomyelin and dihydrosphingomyelin, a mean of 3% of samples were outside this range and excluded from the analyses.

Some individuals had missing covariates for a specific visit. Missing values for the covariates described below ranged from 0% to 20.1% (for APOE genotype only) of the 3972 total visits. As the BLSA follows individuals for decades, we imputed the missing covariates for each individual and visit using information from neighboring visits. This allowed us to utilize the largest number of samples.

We used linear mixed models to account for the longitudinal nature of the data and to model the trajectories for individual sphingomyelin species over time using time-dependent analyses. We used sphingomyelin class and species as the dependent variable. Based on the literature, we initially examined the following predictors: age, age squared, sex, race (white vs. African American), education, the presence of an APOE e4 allele (yes vs. no), BMI, WHR, smoking (never versus ever), depressive symptoms, triglycerides, cholesterol, medications (e.g., statins), and medical conditions (e.g., diabetes, hypertension, CKD), and the interaction term between these predictors and age. We forced age, age squared, sex, and BMI into all models based on the literature and our observations of significant associations with the sphingomyelins in univariate analyses. We then used backward selection with *P *<* *0.10 to determine which variables to include in the final model for each sphingomyelin class and species. All analyses were conducted using STATA Version 11.0 (StataCorp, College Station, TX, USA).

## References

[b1] Bandaru VV, McArthur JC, Sacktor N, Cutler RG, Knapp EL, Mattson MP, Haughey NJ (2007). Associative and predictive biomarkers of dementia in HIV-1-infected patients. Neurology.

[b2] Bandaru VV, Patel N, Ewaleifoh O, Haughey NJ (2011). A failure to normalize biochemical and metabolic insults during morphine withdrawal disrupts synaptic repair in mice transgenic for HIV-gp120. J. Neuroimmune Pharmacol.

[b3] Bandaru VV, Mielke MM, Sacktor N, McArthur JC, Grant I, Letendre S, Chang L, Wojna V, Pardo C, Calabresi P, Munsaka S, Haughey NJ (2013). A lipid storage-like disorder contributes to cognitive decline in HIV-infected subjects. Neurology.

[b4] Contreras FX, Ernst AM, Haberkant P, Bjorkholm P, Lindahl E, Gonen B, Tischer C, Elofsson A, von Heijne G, Thiele C, Pepperkok R, Wieland F, Brugger B (2012). Molecular recognition of a single sphingolipid species by a protein’s transmembrane domain. Nature.

[b5] Deeley JM, Hankin JA, Friedrich MG, Murphy RC, Truscott RJ, Mitchell TW, Blanksby SJ (2010). Sphingolipid distribution changes with age in the human lens. J. Lipid Res.

[b6] Fernandez C, Sandin M, Sampaio JL, Almgren P, Narkiewicz K, Hoffmann M, Hedner T, Wahlstrand B, Simons K, Shevchenko A, James P, Melander O (2013). Plasma lipid composition and risk of developing cardiovascular disease. PLoS ONE.

[b7] Gatt S, Bierman EL (1980). Sphingomyelin suppresses the binding and utilization of low density lipoproteins by skin fibroblasts. J. Biol. Chem.

[b8] Gonzalez-Covarrubias V, Beekman M, Uh HW, Dane A, Troost J, Paliukhovich I, van der Kloet FM, Houwing-Duistermaat J, Vreeken RJ, Hankemeier T, Slagboom EP (2013). Lipidomics of familial longevity. Aging Cell.

[b9] Hammad SM, Pierce JS, Soodavar F, Smith KJ, Al Gadban MM, Rembiesa B, Klein RL, Hannun YA, Bielawski J, Bielawska A (2010). Blood sphingolipidomics in healthy humans: impact of sample collection methodology. J. Lipid Res.

[b10] Hannun YA, Obeid LM (2011). Many ceramides. J. Biol. Chem.

[b11] Haughey NJ, Cutler RG, Tamara A, McArthur JC, Vargas DL, Pardo CA, Turchan J, Nath A, Mattson MP (2004). Perturbation of sphingolipid metabolism and ceramide production in HIV-dementia. Ann. Neurol.

[b12] Haughey NJ, Bandaru VV, Bae M, Mattson MP (2010). Roles for dysfunctional sphingolipid metabolism in Alzheimer’s disease neuropathogenesis. Biochim. Biophys. Acta.

[b13] Hixson JE, Vernier DT (1990). Restriction isotyping of human apolipoprotein E by gene amplification and cleavage with HhaI. J. Lipid Res.

[b14] Holzer M, Trieb M, Konya V, Wadsack C, Heinemann A, Marsche G (2013). Aging affects high-density lipoprotein composition and function. Biochim. Biophys. Acta.

[b15] Jiang XC, Paultre F, Pearson TA, Reed RG, Francis CK, Lin M, Berglund L, Tall AR (2000). Plasma sphingomyelin level as a risk factor for coronary artery disease. Arterioscler. Thromb. Vasc. Biol.

[b16] Kim IC, Lee JH, Bang G, Choi SH, Kim YH, Kim KP, Kim HK, Ro J (2013). Lipid profiles for HER2-positive breast cancer. Anticancer Res.

[b17] Koch W, Ehrenhaft A, Griesser K, Pfeufer A, Muller J, Schomig A, Kastrati A (2002). TaqMan systems for genotyping of disease-related polymorphisms present in the gene encoding apolipoprotein E. Clin. Chem. Lab. Med.

[b18] Kuikka M, Ramstedt B, Ohvo-Rekila H, Tuuf J, Slotte JP (2001). Membrane properties of D-erythro-N-acyl sphingomyelins and their corresponding dihydro species. Biophys. J.

[b19] Mielke MM, Haughey NJ, Bandaru VV, Weinberg DD, Darby E, Zaidi N, Pavlik V, Doody RS, Lyketsos CG (2011). Plasma sphingomyelins are associated with cognitive progression in Alzheimer’s disease. J. Alzheimers Dis.

[b20] Milhas D, Clarke CJ, Hannun YA (2010). Sphingomyelin metabolism at the plasma membrane: implications for bioactive sphingolipids. FEBS Lett.

[b21] Montefusco DJ, Chen L, Matmati N, Lu S, Newcomb B, Cooper GF, Hannun YA, Lu X (2013). Distinct signaling roles of ceramide species in yeast revealed through systematic perturbation and systems biology analyses. Sci. Signal.

[b22] Nelson JC, Jiang XC, Tabas I, Tall A, Shea S (2006). Plasma sphingomyelin and subclinical atherosclerosis: findings from the multi-ethnic study of atherosclerosis. Am. J. Epidemiol.

[b23] Petersen NH, Olsen OD, Groth-Pedersen L, Ellegaard AM, Bilgin M, Redmer S, Ostenfeld MS, Ulanet D, Dovmark TH, Lonborg A, Vindelov SD, Hanahan D, Arenz C, Ejsing CS, Kirkegaard T, Rohde M, Nylandsted J, Jaattela M (2013). Transformation-associated changes in sphingolipid metabolism sensitize cells to lysosomal cell death induced by inhibitors of acid sphingomyelinase. Cancer Cell.

[b24] Quinn PJ, Wolf C (2009). The liquid-ordered phase in membranes. Biochim. Biophys. Acta.

[b25] Schlitt A, Blankenberg S, Yan D, von Gizycki H, Buerke M, Werdan K, Bickel C, Lackner KJ, Meyer J, Rupprecht HJ, Jiang XC (2006). Further evaluation of plasma sphingomyelin levels as a risk factor for coronary artery disease. Nutr. Metab.

[b26] Shimokata H, Tobin JD, Muller DC, Elahi D, Coon PJ, Andres R (1989). Studies in the distribution of body fat: I. Effects of age, sex, and obesity. J. Gerontol.

[b27] Shock NW, Greulich RC, Andres R, Arenberg D, Costa PT, Lakatta EG, Tobin JD (1984). Normal Human Aging: The Baltimore Longitudinal Study of Aging.

[b28] Simons K, Ikonen E (1997). Functional rafts in cell membranes. Nature.

[b29] Simons K, Vaz WL (2004). Model systems, lipid rafts, and cell membranes. Annu. Rev. Biophys. Biomol. Struct.

[b30] Slotte JP, Bierman EL (1988). Depletion of plasma-membrane sphingomyelin rapidly alters the distribution of cholesterol between plasma membranes and intracellular cholesterol pools in cultured fibroblasts. Biochem. J.

[b31] Vaarhorst AA, Beekman M, Suchiman EH, van Heemst D, Houwing-Duistermaat JJ, Westendorp RG, Slagboom PE, Heijmans BT (2011). Lipid metabolism in long-lived families: the Leiden Longevity Study. Age (Dordr).

[b32] Vieira CR, Munoz-Olaya JM, Sot J, Jimenez-Baranda S, Izquierdo-Useros N, Abad JL, Apellaniz B, Delgado R, Martinez-Picado J, Alonso A, Casas J, Nieva JL, Fabrias G, Manes S, Goni FM (2010). Dihydrosphingomyelin impairs HIV-1 infection by rigidifying liquid-ordered membrane domains. Chem. Biol.

[b33] Vouk K, Hevir N, Ribic-Pucelj M, Haarpaintner G, Scherb H, Osredkar J, Moller G, Prehn C, Rizner TL, Adamski J (2012). Discovery of phosphatidylcholines and sphingomyelins as biomarkers for ovarian endometriosis. Hum. Reprod.

[b34] Weir JM, Wong G, Barlow CK, Greeve MA, Kowalczyk A, Almasy L, Comuzzie AG, Mahaney MC, Jowett JB, Shaw J, Curran JE, Blangero J, Meikle PJ (2013). Plasma lipid profiling in a large population-based cohort. J. Lipid Res.

[b35] Yeboah J, McNamara C, Jiang XC, Tabas I, Herrington DM, Burke GL, Shea S (2010). Association of plasma sphingomyelin levels and incident coronary heart disease events in an adult population: Multi-Ethnic Study of Atherosclerosis. Arterioscler. Thromb. Vasc. Biol.

[b36] Yu Z, Zhai G, Singmann P, He Y, Xu T, Prehn C, Romisch-Margl W, Lattka E, Gieger C, Soranzo N, Heinrich J, Standl M, Thiering E, Mittelstrass K, Wichmann HE, Peters A, Suhre K, Li Y, Adamski J, Spector TD, Illig T, Wang-Sattler R (2012). Human serum metabolic profiles are age dependent. Aging Cell.

